# A Narrative Review on the Functional Applications, Safety, and Probiotic Characteristics of *Pichia*

**DOI:** 10.3390/nu17223594

**Published:** 2025-11-17

**Authors:** Fnu Samiksha, Erin Marie D. San Valentin, Grace Li, Maya Blazer, Thomas S. McCormick, Mahmoud Ghannoum

**Affiliations:** 1 Department of Dermatology, Case Western Reserve University, Cleveland, OH 44106, USA; fxs313@case.edu (F.S.);; 2 Center for Medical Mycology, University Hospitals Cleveland Medical Center, Cleveland, OH 44106, USA

**Keywords:** *Pichia*, probiotic yeast, auto-aggregation, co-aggregation, hydrophobicity, epithelial barrier, antimicrobial, antibiotic resistance, immunomodulation

## Abstract

The projected increase in the global probiotics market is driven by growing consumer awareness of gut health and the potential benefits of microbial supplements. However, the market currently exhibits a significant gap in probiotic yeast options, with *Saccharomyces boulardii* being the predominant probiotic yeast available. This limitation restricts diversity and potential tailored applications of alternative probiotic yeast strains. *Pichia*, a genus of yeast, has emerged as a promising candidate. This review explores the safety profile and probiotic attributes of *Pichia* strains, which collectively highlight its potential as an effective probiotic yeast. Evidence indicates that *Pichia* exhibits beneficial characteristics such as resilience in harsh gastrointestinal (GI) conditions, antimicrobial activity against pathogens, and immunomodulatory effects that could contribute to gut health. Additionally, the non-pathogenic nature of *Pichia* and its history of safe and wide use in food and beverage fermentation processes increases confidence in its safety for human consumption. Given its potential, *Pichia* may diversify the probiotic yeast market and present an alternative beneficial organism that may contribute to general health and well-being.

## 1. Introduction

Probiotics are live microorganisms that provide health benefits when taken in sufficient amounts [1]. They are commonly used to improve or restore microbial balance and are administered primarily to support digestive health and manage gastrointestinal (GI) symptoms and disorders [2]. Growing interest in the health applications of probiotics has driven exploration of novel microbial strains with improved resilience and therapeutic efficacy. When selecting probiotic strains, certain standards must be considered to ensure efficacy and safety for human use. In general, candidate probiotic species must be well characterized at the strain level and must have substantial evidence for non-pathogenicity and safety, usually referred to as Generally Regarded as Safe (GRAS) for human consumption [3]. Strains should be able to survive and grow in acidic and bile-rich environments, adhere to the GI tract, and remain viable in adequate doses throughout their shelf life [4]. In practice, both industry and regulatory bodies such as the International Scientific Association for Probiotics and Prebiotics (ISAPP) and food safety authorities (e.g., European Food Safety Authority (EFSA)) further specify that candidate probiotic strains must undergo thorough genomic characterization to confirm identity and exclude acquisition of undesirable genes including virulence factors and resistance. They must also demonstrate functional properties (e.g., adherence, immunomodulatory, or antimicrobial activity) using in both in vitro and in vivo models, and in addition to ensuring safety, should preferably demonstrate efficacy through well-designed human clinical trials. In fact, industry as well as consumers expect that a new probiotic strain should undergo one human clinical trial.

Probiotics comprise both bacterial and yeast-based organisms. Bacterial probiotics have been extensively studied, with the most common being lactic acid bacteria such as *Lactobacillus*, *Bifidobacteria*, and *Lactococcus* species. Their gut health benefits and protective roles against several inflammatory and metabolic disorders have been well documented [5]. However, challenges regarding bacterial viability within the GI tract, particularly the stomach [6], and the risk of transferring antibiotic resistance genes [7] has led yeast-based probiotics to emerge as valuable alternatives. The first probiotic yeast, isolated in the early 20th century, is *Saccharomyces cerevisiae* var. *boulardii* (often referred to as simply *S. boulardii*), and has become widely used since the 1950s for treatment of diarrhea [8]. Recent research has demonstrated the effectiveness of this probiotic yeast strain in addressing a range of GI diseases such as Traveler’s diarrhea, antibiotic-associated diarrhea, Crohn’s disease [9], acquired immunodeficiency syndrome (AIDS)-associated diarrhea, and irritable bowel syndrome (IBS) [10]. As a result of these advantages, non-conventional yeasts with probiotic properties such as strains of the *Pichia* genus are actively being explored to expand the application of probiotic yeasts beyond *S. boulardii* [11]. The growing interest in non-*Saccharomyces* yeasts warrants further exploration to characterize their probiotic potential, mechanisms of action, and safety profiles.

Probiotic yeasts have been gaining attention due to certain advantages as outlined above over bacterial probiotics. Unlike many bacteria, yeasts are naturally resistant to antibiotics, allowing them to survive antibiotic treatment while minimizing the risk of transferring antibiotic resistance genes to pathogens [12]. Yeasts are more resilient against low pH and bile-rich conditions within the GI tract, enhancing their survival and colonization within the gut [13]. Moreover, as eukaryotic microorganisms, yeasts are much larger than bacteria and can act as a steric hindrance against pathogenic bacteria [14]. Various yeast species are also known to produce bioactive compounds with antimicrobial [15], antioxidant [16], and immunomodulatory properties [17]. However, potential risks must also be considered such as potential allergenicity and pathogenicity in immunocompromised individuals. There is also a need for more extensive clinical and mechanistic studies to characterize the safety and therapeutic efficacy of emerging yeast probiotics [18]. The present review aims to gather information regarding different attributes of *Pichia* that support its probiotic potential for human health.

## 2. The Characteristics of *Pichia* as a Genus

### 2.1. Taxonomy and History

**Figure 1:**
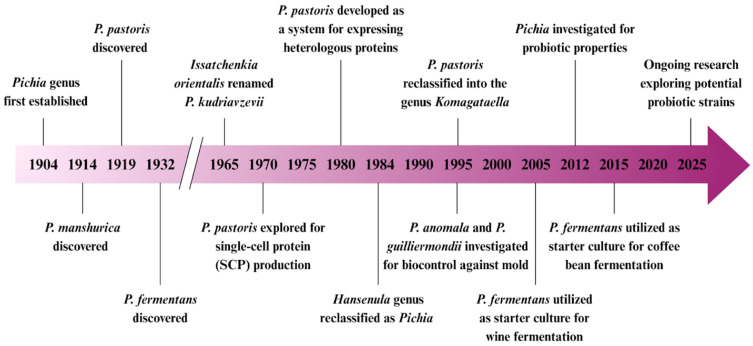
Timeline of taxonomic history and biotechnological advancements of the genus *Pichia*. Organisms within this genus has been extensively utilized in food and beverage production, with recent recognition of its probiotic potential driving renewed scientific interest.

The history of the genus *Pichia* begins in 1904 when Hansen first established the genus based on distinct morphological characteristics (Figure 1). Early key species identified were *Pichia manshurica* (1914) [19] and *Pichia pastoris* (1919) [20]. The genus continued to expand with the discovery of *Pichia fermentans* in 1932 and the reclassification of *Issatchenkia orientalis* as *Pichia kudriavzevii* in 1965 [19]. The 1970s marked a turning point for *Pichia pastoris,* which was explored for its potential in single-cell protein production and gained prominence as a system for expressing heterologous proteins, becoming a valuable workhorse in biotechnology in the 1980s [21]. Taxonomic refinements occurred in 1984 when the genus *Hansenula* was reclassified under *Pichia* [22]. Later, in 1995, *P. pastoris* was reclassified into the genus *Komagataella* [20]. That same year, *Pichia anomala* and *Pichia guilliermondii* began to be investigated for their biocontrol applications against mold [23]. By 2005, *P. fermentans* was applied as a starter culture in wine fermentation, further strengthening the diverse applications of *Pichia* species across food and biotechnology sectors [24].

*Pichia* has traditionally been distinguished by its unique modes of asexual and sexual reproduction and by characteristic morphological features such as multilateral budding and the production of spheroidal, hat-shaped, or hemispheroidal ascospores. In asexual reproduction, *Pichia* undergo multilateral budding, which distinguishes its budding behavior from other genera [25]. Pseudohyphae occurrence, while occasional, is also an identifier of the *Pichia* genus [19,26]. During sexual reproduction, one to four ascospores can be found in unconjugated asci, which may be either persistent or deliquescent [25]. *Pichia* can be identified through ascospore morphology, which can be either spheroidal, hat-shaped, or hemispheroidal, and may or may not have a ledge [19,26]. Currently, the genus *Pichia* is taxonomically classified under subphylum Saccharomycotina, class Pichiomycetes, order Pichiales and family Pichiaceae [27]. Its phylogenetic classification has been widely discussed, with major taxonomic reclassifications occurring following the emergence of DNA analysis tools, which revealed that traditional morphological characters often failed to delimit monophyletic groups. Many species previously assigned to other genera, such as *Saccharomyces* and *Willia,* are now classified under the *Pichia* genus [25]. Similarly, many species formerly placed in *Pichia* based on ascospore morphology and multilateral budding have since been transferred to newly circumscribed genera such as *Wickerhamomyces*, *Meyerozyma*, *Barnettozyma*, *Ogataea*, and *Candida*, resulting in a more narrowly defined *Pichia* [27].

The *Pichia* genus can also be distinguished through genomic analysis. The translation elongation factor-1a (*Tef-1a*) gene, as well as large and small subunit rRNA genes, can accurately determine whether an organism belongs to *Pichia* [26,28]. In a recent study, Zhu et al. [29] used the D1/D2 domains in the large subunit rRNA gene and the ITS region to reclassify species under the *Candida* genus to the *Pichia* genus. In addition to genomic markers, functional traits can also be used as indicators of *Pichia*. Species within the *Pichia* genus are able to ferment glucose [19,26], but cannot assimilate nitrate and seldom ferment other sugars [25].

Within the *Pichia* genus, there are 41 known species at the time of writing [26]. This genus is notable for its adaptability, with species isolated from a wide range of environments [30]. For example, *Pichia* has been found in diverse habitats such as marine sediment [26,29], bark [26], decayed wood, soil [31], flowers [32], insect larvae gut [33], rotting wood [34], columnar cacti necrotic tissue [35], and deteriorated strawberry soft drinks [26,29]. Geographically, *Pichia* species have been isolated in multiple countries including China [36], Columbia, Brazil, UK [29], Japan [37], Mexico [38], Thailand [39], Indonesia [31], The Netherlands [40], India [41], Caribbean [35], Spain [42], and Borneo [32]. As for their ecological tolerance, *Pichia* species can survive at temperatures of up to 42 °C [43]; however, one *P. kudriavzevii* strain has been found to survive at 45 °C [43]. Ecologically, some species act as plant endophytes, where their antifungal properties contribute to the plant’s defense responses [30,44]. For example, *P. kudriavzevii* isolated from Golden Delicious apples inhibits the growth of the fungal pathogen *Botrytis cinerea*, effectively controlling gray mold disease [30].

### 2.2. Historical Uses of Pichia Species

Different *Pichia* species have had varying scientific and industrial uses depending on their properties. Common *Pichia* species that have been utilized in the fields of biotechnology and molecular biology include *Pichia kluyveri*, *P. fermantans*, *P, anomala*, *Pichia stipitis*, *P. pastoris*, and *P. kudriavzevii* [25]. *P. pastoris* is commonly used for synthesizing recombinant proteins because of its effective and versatile system [45,46]. The methylotrophic yeast can accurately fold proteins, undergo glycosylation, and form disulfide bonds, thus ensuring proper functionality of expressed proteins [46]. It has a co-translational expression system and can secrete recombinant proteins into the supernatant [46,47,48]. Furthermore, the genetic alterations of *P. pastoris* are tractable, possess strong promoters, and can be cultivated to high cell densities, facilitating large-scale protein production. Recombinant *P. pastoris* proteins are used to produce cellulase, an important enzyme that degrades cellulosic biomass for biofuel production. Additionally, *P. pastoris* is utilized across the industrial and pharmaceutical sectors to produce enzymes such as xylanases, lipases, xanthophylls, and glucaric acid [46,49].

Several *Pichia* species, including *P. anomala*, *P. fermentans*, *P. guiliermondii*, *P. kudriavzevii*, *P. pastoris*, *P. membranifaciens*, and *P. stipitis* are commonly used in winemaking due to their capacity to enhance aroma and ethanol yield [25,50]. Among them, *P. kluyveri* is widely studied for its commercial applications in the fermentation process of no-alcohol and low-alcohol beer (NALAB) and kombucha [51,52]. *P. kluyveri* produces low amounts of ethanol due to its inability to metabolize complex sugars, only metabolizing monosaccharides [51,53]. In NALAB fermentation, *P. kluyveri* is being used in the production process due to its high production of isoamyl acetate, which results in a fruity-banana flavor to the beverage [51,54]. Similarly, *P. kluyveri* can also produce a fruity flavor to kombucha as the yeast accelerates the kombucha fermentation process [52]. Collectively, the *Pichia* genus demonstrates proven utility both in the context of research and commercial applications.

## 3. Regulatory and Safety Profile of *Pichia*

In probiotic development, safety is a paramount necessity, and regulatory frameworks are crucial for directing microbial usage in food and feed. Several *Pichia* strains have been used in the food and beverage industry, often with self-declared GRAS status. For example, *P. pastoris*, *P. anomala*, and *P. kluyveri* have been widely used in wine fermentation. At the time of writing, *P. kluyveri* DSM 33235 is the only *Pichia* strain to be granted GRAS status, primarily for use as a starter culture in the production of alcohol-free and low-alcohol beer, as well as in fermented vegetables, fruit juices, and tea for flavor and aroma enhancement [55]. The FDA concluded that there were no questions regarding the safety of *P. kluyveri* DSM 33235 under its intended use, based on its history of safe presence in various fermented foods, non-pathogenic profile, and absence of toxin production.

In Europe, the European Food Safety Authority (EFSA) awards Qualified Presumption of Safety (QPS) status, which is a system for considering the safety of microorganisms approved for use in the food chain [56]. The QPS list is based on a rigorous review of critical criteria such as taxonomic clarity, complete scientific understanding, lack of pathogenicity, and a track record of safe use. Several species of the genus *Pichia*, including *Pichia jadinii*, *Pichia hansenii*, *P. pastoris*, *Pichia angusta*, and *P. anomala*, have been included to the updated EFSA QPS list [57]. This validation highlights their established importance in various traditional fermentation techniques and food biotechnology, as well as a significant step forward in their transition to probiotic applications. The EFSA’s recognition confirms their general safety and encourages the use of *Pichia* as a probiotic yeast, especially given its established functional features like GI survival, stress resistance, and antibacterial activity. The QPS status thus provides a solid regulatory base, increasing the practicality of adding *Pichia* strains into functional food products and broadening the probiotic repertoire beyond traditional bacteria and *Saccharomyces* species. However, not all species within the *Pichia* genus qualify for this status. In particular, *P. kudriavzevii* (syn. *Candida krusei*), although frequently studied for its promising probiotic features is excluded from both GRAS and QPS due to known safety risks, particularly in immunocompromised individuals [58,59]. Clinical and environmental isolates of *P. kudriavzevii* are genetically identical and both consistently exhibit high resistance to fluconazole [43,60]. *P. kudriavzevii* is recognized as one of the *Candida* spp. that cause systemic infections resulting in morbidity and mortality in hospital settings [59,61]. These findings highlight the necessity for careful strain selection and rigorous safety and resistance evaluation prior to it being used as a probiotic or commercially deploying *Pichia* strains, with particular emphasis on avoiding use in high-risk individuals and close monitoring for opportunistic infections.

## 4. Probiotic Attributes of *Pichia*

Given the robust status of yeast-based probiotics, identifying and characterizing novel yeast strains with probiotic potential is desirable for expanding their applications. While *S. boulardii* is currently the most widely used and studied probiotic yeast, yeasts such as *Pichia* species have also shown promising probiotic properties including high tolerability in GI environments, antimicrobial and antioxidant activity, and immunomodulatory effects.

### 4.1. Gastric Survival and Bile Tolerance

Probiotic microorganisms encounter various environmental conditions upon ingestion by the host and during transit in the GI tract. To be an effective probiotic, microorganisms must be able to demonstrate resistance to two critical physiological barriers. Firstly, they need to tolerate conditions of the stomach where pH may decrease to as low as 1.5 to 3.5 and fluctuate to pH 3 to 5 during food intake [62]. Acid tolerance for at least 90 min is a preferable trait for probiotic supplements [63].

**Figure 2:**
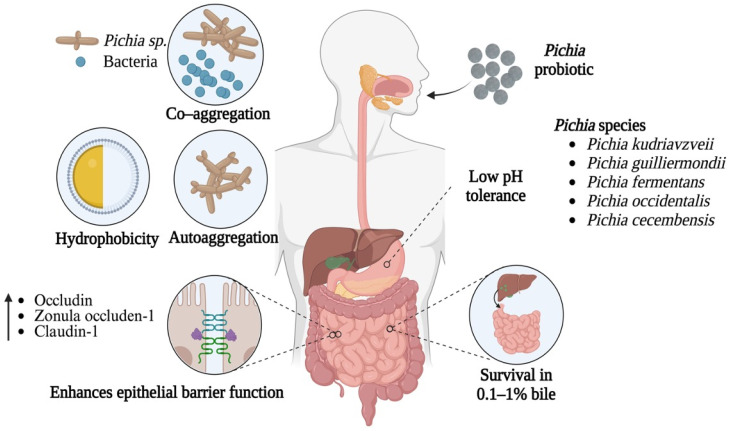
Favorable factors found in *Pichia* that contribute toward probiotic potential. The schematic highlights important characteristics of *Pichia* that facilitate survival including low pH tolerance, bile acid resistance, and the ability to adhere, auto-aggregate, and interact beneficially within host environments, supporting their potential as probiotic agents. The use of *Pichia* as a probiotic also enhances epithelial barrier function by upregulating tight junction proteins. Created using BioRender.com.

Bile tolerance represents a second barrier. The small intestines contain bile secreted from the liver and pancreatic juice to aid digestion [64], with the optimal bile concentration ranging from 0.2% to 0.6% [65]. Bile salts possess detergent properties, which can damage the human GI tract and exert antimicrobial effects on yeast [66]. Therefore, resistance to both gastric acid and bile conditions represent critical criterion for *Pichia* to successfully navigate the GI landscape (Figure 2).

A number of *Pichia* species have been reported to survive in low pH and tolerate bile conditions (Table 1). Wang et al. [67] isolated an efficient acetic acid-tolerant *P. kudriavzevii* Y2 from the water of *baijiu* brewing waste. This strain was reported to tolerate 12 g/L of acetic acid, which has a pH of approximately 2.72. In another study, two *P. kudriavzevii* strains, NBRC1279 and NBRC1664, were cultured and compared for growth in highly acidic conditions (SCD medium containing 15 mM formic acid, 35 mM sulfuric acid, 60 mM hydrochloric acid, 100 mM acetic acid, or 550 mM lactic acid), where only strain NBRC1664 could tolerate low pH conditions [68]. *P. kudriavzevii* isolated from the gut of *Pila globosa* (an edible freshwater snail) also showed strong gastric survival (pH 1.5–10) and bile tolerance (1.2%), together with bile salt hydrolase (BSH) activity, supporting its survival in the GI tract [69]. In another study by Lata et al. [70], it was shown that *P. kudriavzevii* Y33 isolated from traditional mango pickle maintain 88.6% viability at pH 2 and over 95% in 2% bile, suggesting excellent gastric and bile tolerance under harsh GI conditions. Helmy et al. [71] observed *P. kudriavzeviil* QLB isolated from Karish cheese showed tolerance to bile concentration of 2%. In another work by Menezes et al. [62], one strain of *P. membranifaciens* and *P. guillermondii* and two strains of *P. kudriavzevii* survived in the GI tract by tolerating the acidic pH in gastric conditions and bile salts.

Lucena et al. [72] demonstrated that yeasts have been able to modify the components of their cell walls for survival in low pH levels by activating the cell wall integrity pathway. A study by Fletcher et al. [73] demonstrated that *P. anomala* has low acid tolerance due to its strong expression of H^+^-ATPases in the plasma and vacuolar membranes which expel protons to maintain cytosolic pH balance. This process is aided by increased mitochondrial respiration, including the expression of Complex I subunits, allowing for more ATP generation. Unlike *S. cerevisiae*, *P. anomala* respires rather than ferments during stressful conditions, resulting in increased biomass yield and growth at low pH. Together, these strategies enable *P. anomala* to thrive and survive in low acidic conditions.

### 4.2. Cholesterol Assimilation

Another important health benefit attributed to *Pichia* is its well-documented ability to assimilate and sequester cholesterol from the surrounding environment. Lowering cholesterol levels is critical to prevent coronary artery disease and previous use of probiotic bacteria to reduce serum cholesterol levels has attracted attention [74]. Similarly, yeasts are also known to assimilate cholesterol through different mechanisms. Lata et al. [70] demonstrated the physical incorporation of cholesterol into the cell membrane during growth. This process reduces cholesterol levels in the surrounding environment without metabolizing it as a carbon source. On the other hand, bile salt hydrolase (BSH) produced by *Pichia* also plays a role in promoting bile salt detoxification and enhancing cholesterol elimination through increased fecal bile acid secretion [69]. Alkalbani et al. [14] proposed an additional mechanism involving the attachment of cholesterol to the yeast cell wall, enzymatic reduction of cholesterol to coprostanol, and the disruption of cholesterol micelles facilitated by BSH activity.

Studies on *P. kudriavzevii* have reported cholesterol assimilation rates ranging from approximately 40% to over 97% (Table 1), depending on the substrate and experimental conditions. *P. kudriavzevii* isolated from the gut of freshwater snail showed a moderate cholesterol assimilation rate of around 20%, alongside other probiotic properties such as BSH activity and pathogen inhibition, implying its potential as a cholesterol-lowering probiotic [69]. Another strain, *P. kudriavzevii* O21, demonstrated exceptionally high cholesterol assimilation rates (95.02% ± 1.43%) in vitro [75]. Importantly, in vivo evidence reinforces these in vitro findings. Supplementation with a *P. kudriavzevii*-fermented cereal mix in mice fed a high-cholesterol diet significantly reduced serum total cholesterol, triglycerides, and low-density lipoprotein (LDL), and increased high-density lipoprotein (HDL) [76]. The improved lipid profile corresponded with a notably lower atherogenic index, suggesting the potential role of *Pichia* as a functional probiotic yeast in supporting cardiovascular health through cholesterol assimilation.

**Table 1:** Probiotic characteristics of selected *Pichia* species, including physiological traits, resistance to gastrointestinal conditions, and potential mechanisms of action underlying their beneficial effects.

Strain	Origin	Cholesterol Assimilation	Survival pH	Survival Bile	Optimal Growth Temperature	Auto Aggregation	Reference
*P. cecembensis* AA19	Addis Ababa	89.0%	95.77% survival at pH 2	90.43% survival at 0.3% bile	15 °C and 37 °C	69.36–89.39% (after 24 h)	[77]
*P. kudriavzevii* HJ2	Marine medicinal mangroves	79.98% degradation at 24 h	2.0 (83.28% biomass at 24 h)	3% bile (81.25% biomass at 12 h)	Tolerates extreme temperatures	92.41% at 180 min	[78]
*P. kudriavzevii* BY10	Raw milk (China)	43.2% at 72 h	Survives at pH 1.5, 2.0, 3.0, 5.0	16.1% survival at 0.5% bile	Not reported	High adhesion (62 cells/100 HT-29)	[79]
*P. kudriavzevii* BY15	Raw milk (China)	44.4% at 72 h	Survives at pH 3.0, 5.0; viable at pH 2	18.9% at 0.5% bile	Not reported	Moderate adhesion	
*P. fermentans* BY5	Raw milk (China)	40.3% at 72 h	pH 3.0 and 5.0	9.5% at 0.5% bile	Not reported	Moderate adhesion	
*P. guilliermondii* BY31	Raw milk (China)			81.4% at 0.5% bile		Moderate	
*P. kudriavzevii* Y33	Traditional home-made mango pickle	High cholesterol assimilation of bile and taurocholate, at 88.58 and 86.83%, respectively	88.62% survival at pH 2	95% at 2% of bile		High auto-aggregation ability of 87% after 24 h and 72.45% after 3 h of incubation	[70]
*P. kudriavzevii* OG32	Cereal-based functional food (fermented)	Reduced serum TC, TG, LDL-C; increased HDL-C; lowered atherogenic index	Tolerated gut-like pH	Tolerated bile-rich diet	Survived in rat gut (37 °C)	Not reported	[76]
*P. kudriavzevii* YGM091	Fermented goat milk	Not directly reported; potential implied via bile tolerance	Survived pH 2.0 (80.73%) and pH 3.0 (247.13%) after 3 h	Tolerated 2.0% bile salts with >110% survival after 5 h	Grew well at 25 °C, 37 °C, and 42 °C (growth at all tested temperatures)	88.64% at 60 min; 89.91% at 90 min	[80]
*P. kudriavzevii* OG32	Ogi (Nigeria)	74.05% at 48 h	Survives pH 2.0 (100% at 3 h)	100% survival in 2% bile (3 h)	Grows at 37 °C (μ = 0.29 h^−1^)	91.85% at 24 h	[81]
*P. guilliermondii* CCMA 1753	Fermented table olives (Brazil)	Not reported	Survived pH 2.0 for 3 h (92.23%)	Survived 0.3% bile for 3 h (92.73%)	37 °C (tested temp)	Intermediate (~65%)	[82]
*P. kudriavzevii* QAUPK01	Human feces	66.7%	7.2	Tolerant	37	Not reported	[83]
*P. kudriavzevii* QAUPK02	Human feces	68.2%	7.2	Tolerant	37		
*P. kudriavzevii* QAUPK03	Human feces	83.6%	7.2	Tolerant	37		
*P. kudriavzevii* QAUPK04	Human feces	79.3%	7.2	Tolerant	37		
*P. kudriavzevii* QAUPK05	Human feces	85.2%	7.2	Tolerant	37		
*P. kudriavzevii* GBT37	Dadih (West Sumatra)	High (exact % not stated)	pH 2–6		37 °C	Not specified	[84]
*P. occidentalis* GBT30							
*P. kudriavzevii* M26, M28, M29, O9, G6, G5, M30, M31	Fermented cereal foods (African origin)	Not reported	Tolerated pH 2 (≈31% of isolates survived this stress)	Tolerated 0.3% bile (≈99% of isolates)	Tolerated pH 2 (≈31% of isolates survived this stress)	12.7–40.9%	[85]
*P. kudriavzevii* O21	Fermented dairy/non-dairy product	91.5%	High (OD ~0.85)	Oxgall: ~85%, Cholic: ~89%, Taurocholic: ~91%	Tolerated 60 °C for 5 min	~85%	[14]
*P. kudriavzevii* O26		92.3%	High (OD ~0.83)	Oxgall: ~88%, Cholic: ~90%, Taurocholic: ~90%	Tolerated 60 °C for 5 min	~83%	
*P. kudriavzevii* SH55		96.5%	High	~90.5% (24 h, mixed bile salts)	Tolerated 60 °C for 5 min	~84%	
*P. kudriavzevii* O12		87.2%	High	~87.4% (oxgall), ~86% (others)	Tolerated 60 °C for 5 min	~78%	
*P*. *kudriavzevii* KT000037/URCS7	Cryopreserved food sample	Not directly tested	pH 1.5–11; Survives >70% at pH 2 for 120 h	0.1%: 99%; 0.3%: 84%; 0.5%: 70% after 4 h at 37 °C	Survives 95 °C (2 h), 121 °C (15 min)	59.12% (5 h), 81.23% (24 h)	[86]
*P. kudriavzevii* YGM091	Fermented goat milk	Not specified	pH 3.0 (247.13%), pH 2.0 (80.73%)	0.5% (145.03%), 1% & 2% bile (up to 110%)	25, 37, 42 °C	66.56% after 45	[80]
*P. kudriavzevii* MYSSBYPS10	Fermented green gram dosa batter	Not reported	pH 2.0 83.79% survival at 2 h 68.31% at 4 h	0.3% bile 92.95% survival at 2 h 89.65% at 4 h	Broad temp range	97.2% at 24 h	[12]

### 4.3. Auto-Aggregation, Co-Aggregation, and Hydrophobicity

Optimal probiotics must not only survive the upper digestive system but also adhere to host intestinal cells to facilitate colonization and proliferation in the intestinal tract [87,88]. Common properties of probiotic strains that encourage colonization include auto-aggregation, co-aggregation and hydrophobicity, required for attachment to intestinal epithelial cells [89]. It has been observed that auto-aggregation with a potential of more than 80% is considered durable and strains having high hydrophobicity exhibit upright (more durable) adhesion to intestinal cell lines [90]. Microbial adhesion to hydrocarbons has been widely used to measure the cell surface hydrophobicity of probiotics. In vitro evaluation of auto-aggregation and ability to co-aggregate with potential enteric pathogens has been used previously as a preliminary screening and selection tool for probiotics [11,91]. These important probiotic properties in different *Pichia* species have been observed in a number of studies; *P. kudriavzevii* [11,12,14,80,86,92,93], *P. stutzeri* XL-2 [94], *P. fermentans* [93], *P. cactophila*, *P. jadinii* [95], *P. barkeri* VIT-SJSN01 [90], and *P. guilliermondii* CCMA 1753 [82], each indicating that various *Pichia* species exhibit efficient auto-aggregation, hydrophobicity, and co-aggregation with pathogens.

For example *P. kudriavzevii* isolated from *Pila globosa* demonstrated high auto-aggregation (93%) and hydrophobicity (76.9%), suggesting a strong adhesion to intestinal epithelial cells, which increased protection by forming a physical barrier against pathogens [69]. In another study, *Pichia* sp. DU2 (similar to *Pichia cactophila* ~99.67%) showed co-aggregation (21.8–46.78%), auto-aggregation (18.21 to 71.92%), and hydrophobicity-like (43.3%) characteristics implying the probiotic potential of *Pichia* [96]. Merchan et al. [95] reported different probiotic attributes of *P. cactophila* strains which were isolated from traditional soft cheese, like survival in acidic conditions (pH 2.5), tolerating bile salts (0.3%), auto-aggregation (38.75–50.83%), and hydrophobicity (33.07–55.11%). The co-aggregation ability of *Pichia kluyveri* has been demonstrated in a study by Yildiran et al. [97] where it co-aggregated with *S. aureus*, *E. coli*, *Listeria monocytogenes*, and *Salmonella typhimurium.* Collectively, these findings emphasize the auto-aggregation, co-aggregation, and hydrophobicity potential of different *Pichia* species from diverse sources, implying their promising role as probiotic.

### 4.4. Epithelial Barrier Function

Intestinal epithelial cells form a monolayer that serves as a physical barrier between the host’s immune system and the external environment of the gut lumen [98]. Tight junctions (TJ), adherent junctions, gap junctions, and desmosomes secure the integrity of this epithelial barrier. TJs are located towards the apical side of the intestine and are made up of transmembrane proteins such as occludin, claudin, and junctional adhesion molecules, which all interact extracellularly with different comparable TJ proteins in neighboring cells, and intracellularly with the cell’s own cytoskeleton via zonula occludens (ZO) proteins and filamentous actin [99]. Various chronic inflammatory diseases, such as inflammatory bowel disease (IBD), have been linked to TJ integrity loss [100].

Numerous studies have demonstrated that *Pichia* species can regulate the function of TJ in various organisms. It has been shown that selenium-enriched *P. kudriavzevii* (HSeY) strengthened intestinal barrier function in murine models by increasing goblet cell numbers, upregulating MUC2 expression, and enhancing expression of tight junction proteins like ZO-1, claudin-1, and occludin, thus alleviating dextran sodium sulfate (DSS)-induced colitis [92]. HSeY also alleviated gut microbiota dysbiosis by promoting the colonization of beneficial bacteria such as *norank-f-Muribaculaceae* and *Bacteroides* while suppressing harmful microorganisms such as *norank-f-norank-o-Clostridia*-UCG-014. This probiotic strain and its cell-free supernatant have also been reported to considerably increase the mRNA expression level of zonulin-1, occludin-1 and claudin-1 in lipopolysaccharide (LPS) challenged Caco-2 cells, suggesting a protective effect on the intestinal barrier [101]. *P. manshurica* also showed promising probiotic potential by considerably reducing the association of *Salmonella enteritidis* with intestinal epithelial cells (Caco-2/TC-7), where pretreatment with *P. manshurica* decreased pathogen adhesion by 30% possibly through a barrier effect [91]. In addition, co-culture of *Pichia* with the *S. enteritidis* led to an even greater reduction (67–82%) in its attachment to cells, emphasizing the importance of live yeast cells. Zhang et al. [102] found that by upregulating the expression of TJ proteins like occludin, ZO-1, and claudin-1, modified *P. pastoris* expressing surface-displayed pectinase PG5, a subtype of pectinase that degrades pectin into beneficial pectin oligosaccharides, enhanced gut barrier function. In vivo, the production of these oligosaccharides promotes mucosal healing and reduces inflammation. Compared to the free enzyme, whole-cell pectinase PG5 exhibited superior enzymatic stability and activity within the GI tract, resulting in more effective restoration of epithelial integrity in DSS-induced colitis models. *P. anomala* AR2016 had a favorable effect on gut barrier function as it promotes the expression of ZO-1 and occludin. The strain also upregulates the expression of alkaline phosphatase (ALP), which dephosphorylates bacterial LPS and reduces inflammation. Furthermore, *P. anomala* also reduces hazardous amino acid decarboxylase activities like histidine decarboxylase, lysine decarboxylase and tryptophan decarboxylase, which are linked to diarrhea and gut toxicity. These combined effects contribute to healthier intestinal barriers, particularly during stress [103]. These findings indicate that distinct *Pichia* species can play immunomodulatory and protective roles in the context of host organisms by altering the integrity and function of TJs, particularly in the gut. This supports *Pichia*’s potential use in supplements aimed at improving gut health.

### 4.5. Antimicrobial Properties

Suppressing the growth of pathogenic organisms is considered one of the most important characteristics of probiotic microorganisms. Yeasts are known to inhibit pathogens from binding to enterocytes by exerting a direct antagonistic impact and/or secreting different metabolites and enzymes for survival. *S. boulardii* was previously shown to compete with pathogenic microbes for food and mucosal receptors in the GI tract, thus preventing pathogens from colonizing and dominating the gut of the host [104].

Similarly, various *Pichia* species have demonstrated strong antimicrobial activities against a range of pathogenic organisms (Table 2). Lata et al. [70] reported that *P. kudriavzevii* Y33, isolated from traditional homemade mango pickle, exhibited significant inhibition zones against *Salmonella typhi, Escherichia coli, Shigella, Pseudomonas aeruginosa, Bacillus cereus*, *Staphylococcus aureus*, *Aeromonas hydrophila*, and *L. monocytogenes*. Other strains, such as *P. guilliermondii* CCMA 1753 from Brazilian table olive fermentation [82], *P. kudriavzevii* GBT37 and *Pichia occidentalis* GBT30 from Dadih (West Sumatra) [84], and *Pichia norvegensis* WSYC 592 [105] from dairy products have also shown varying degrees of antibacterial activity, particularly against *Staphylococcus aureus*, *L. monocytogenes*, and *B. cereus*. *P. fermentans* and *P. anomala* have produced anti-listerial peptides that reduce *Listeria* populations in cheese models. Multiple strains of *P. kudriavzevii* isolated from fermented dairy and non-dairy foods exhibit strain-dependent antimicrobial effects on pathogens such as *E. coli* O157:H7, *S. aureus*, *S. typhimurium*, and *L. monocytogenes*. Other notable strains include *Pichia cecembensis* AA19 from Ethiopia, *P. kudriavzevii* KT000037 [86], and *P. pastoris* X-33 used as a feed additive, all reported to inhibit various human and animal pathogens. Moreover, *P. kudriavzevii* YGM091 from fermented goat milk and *P. kudriavzevii* MYSSBYPS10 from fermented green gram dosa batter demonstrated significant inhibition of pathogens and phytopathogens, respectively. *P. kudriavzevii* C-1 from traditional Kazakh dairy products [106] and *P. kudriavzevii* MH458240 (M9) from fermented ogi in Nigeria [107] also showed antimicrobial activity against a range of pathogens. Utama et al. (2021) [108] studied the antifungal and aflatoxin-reducing activity of β-glucan isolated from *P. norvegensis* grown on tofu wastewater and reported that *P. norvegensis* and its β-glucan showed an inhibition zone against *Aspergillus flavus*.

**Table 2:** Reported antimicrobial activities and measurement types for different *Pichia* species.

Strain	Origin	Target Organism	Inhibition (mm)	Reference
*P. kudriavzevii* Y33	Traditional home-made mango pickle	*S. typhi*	10.50 ± 1.50	[70]
		*E. coli*	10.00 ± 2.00	
		*Shigella*	13.50 ± 1.50	
		*P. aeruginosa*	14.00 ± 1.00	
		*B. cereus*	12.50 ± 0.50	
		*S. aureus*	13.50 ± 0.50	
		*A. hydrophilla*	22.00 ± 2.00	
		*L. monoctyogenes*	10.50 ± 1.50	
*P. guilliermondii* CCMA 1753	Table olive fermentation (Brazil)	*S. aureus*	<10	[82]
		*S. enteritidis*	Between 20 and 30	
		*L. monocytogenes*	Between 20 and 30	
*P. kudriavzevii* GBT37	Dadih (West Sumatra)	*B. cereus*	4.55 ± 0.20	[84]
		*S. aureus*	4.20 ± 0.33	
		*EPEC K.1.1*	3.68 ± 0.27	
		*Listeria* sp.	5.50 ± 0.19	
*P. occidentalis* GBT30		*B. cereus*	2.50 ± 0.17	
		*S. aureus*	3.40 ± 0.32	
		*EPEC K.1.1*	4.00 ± 0.25	
		*Listeria* sp.	7.67 ± 0.17	
*P. cecembensis* AA19	Addis Ababa Ethiopia	*E. coli*	19	[77]
		*S. aureus*	17	
		*Salmonella typhi*	15	
		*B. cereus*	18	
*P. kudriavzevii* KT000037	Xylose-utilizing yeast from cryopreserved food sample	*E. coli*	13	[86]
		*S. aureus*	19	
		*E. faecalis*	26	
		*M. luteus*	22	
		*K. pneumoniae*	21	
		*S. typhi*	22	
		*P. aeruginosa*	22	
		*S. paratyphi B*	23	
		*P. mirabilis*	21	
		*V. cholerae*	19	
		*S. flexneri*	17	
*P. kudriavzevii* C-1	Traditional Kazakh dairy product	*E. coli*	11	[106]
		*S. aureus*	15	
**Studies with inhibition expressed as %**				
*P. pastoris* X-33	Feed additive	*S. typhimurium* in LB	43	[109]
		*S. typhimurium* in YPD	86	
*P. kudriavzevii* YGM091	Fermented goat milk	*E. coli*	88.75	[80]
		*S. aureus*	88.34	
		*S. typhimurium*	79.4	
*P. kudriavzevii* MYSSBYPS10	Fermented green gram dosa batter	*M. phaseolina*	69.41	[12]
		*A. niger*	64.72	
		*F. oxysporum*	68.6	
*P. anomala*	Not specified	*C. gloeosporioides*	79.63	[110]
**Qualitative Studies**				
*P. kudriavzevii* G1, O12, O13, O21, O26, O36, 066, SH40, SH45	Fermented dairy and non-dairy foods	*E. coli* O157:H7 *S. aureus* *S. typhimurium* *L. monocytogenes*	Strong to moderate (strain-dependent)	[75]
*P. kudriavzevii* MH458240 (M9)	Fermented ogi (Nigeria)	*E. coli* *Pseudomonas* sp. *S. aureus* *Klebsiella* sp. *Proteus* sp.	Not reported (qualitative)	[107]
*P. kudriavzevii* MH458239 (M5)				
*P. norvegensis* WSYC 592	Dairy product (milk)	*Listeria*	7-log reduction in co-culture and ~1.5 log on Tilsit cheese	[105]
*P. fermentans*	Dairy product (cheese)	*Listeria*	Reduced *Listeria* by ~3-log in Camembert curd model	[15]
*P. anomala* (*Wickerhamomyces anomalus*)	Dairy (Camembert curd)	*Listeria*	Produced heat-stable anti-listerial peptides; caused pore formation and bacterial cell lysis	
*P. farinosa*	Human oral cavity	*Candida albicans*	Nutrient competition and protein-mediated virulence inhibition.	[111]
		*Aspergillus*		
		*Fusarium*		

A more comprehensive study on the antimicrobial properties of *Pichia* has been shown in a study by Mukherjee et al. [111]. An inverse relationship between *Pichia* and *Candida* abundance was initially observed in the oral microbiome of HIV-infected individuals, suggesting a natural antagonism. Further in vitro experiments confirmed that *Pichia* spent medium (PSM), the fluid remaining after culturing *Pichia*, significantly inhibited the growth of *Candida*, *Aspergillus*, and *Fusarium*. PSM also suppressed vital *Candida* pathogenic traits such as biofilm formation, germination, and adhesion to surfaces. The antifungal effect appeared to result from nutrient competition and secreted heat-stable, non-glycosylated proteinaceous factors. A candidiasis mouse model was also used to show that topical PSM treatment dramatically reduced infection severity and fungal burden (*p* ≤ 0.029), even showing better results compared to the polyene antifungal nystatin-treated controls.

These findings emphasize the broad-spectrum antimicrobial potential of different *Pichia* species and strains derived from diverse fermented foods, underscoring their promising role as probiotic agents in combating pathogenic bacteria.

### 4.6. Antibiotic Resistance

To function properly, probiotics must be viable and capable of generating an equilibrium within the host GI tract [112]. Yeasts, in particular, are advantageous in this context as they are innately resistant to bactericidal antibiotics, which is a trait common to all fungi [113]. This antibiotic resistance is a beneficial characteristic for probiotic organisms since it allows them to withstand antibiotic treatments that typically kill beneficial bacterial probiotics. Unlike antibiotic-resistant *Lactobacillus* strains, which can transfer resistance genes to harmful bacteria [7,114], horizontal gene transfer between yeasts and bacteria is highly unlikely [115,116]. This property favors yeast-based probiotics like *Pichia* during or following antibiotic therapy. Numerous studies have reported the antibiotic-resistant properties of *Pichia* species. For example, Wang et al. [117] evaluated the probiotic potential of yeast strains *P. kudraivzevii* GBY1 and *S. cerevisiae* GBY2 isolated from kombucha in New Zealand and demonstrated that the yeast were resistant to eight tested antibiotics (ampicillin, chloramphenicol, colistin sulfate, kanamycin, nalidixic acid, nitrofurantoin, streptomycin, and tetracycline), with high levels of antioxidant activities (>90%). Lata et al. [70] evaluated the probiotic potential of *P. kudriavzevii* Y33 isolated from traditional home-made mango pickle and reported that it was resistant to different antibiotics including vancomycin, penicillin, clindamycin, and ampicillin. In another study by Kathade et al. [69], it was demonstrated that *P. kudriavzevii* was resistant to ampicillin (10 mcg), chloramphenicol (25 mcg), streptomycin (10 mcg), sulphatriad (300 mcg), tetracycline (25 mcg), and penicillin-G (1 unit) as per the interpretation of zones of inhibition for Kirby-Bauer antibiotic susceptibility testing.

### 4.7. Immunomodulatory Properties

Immunomodulation is one of the modes of action used by probiotic yeasts to control pathogens. It has been reported that β-glucans ((1→3)-β-d-linked polymers of glucose) present in yeast cell walls have modulating effects on innate immune cell activity and cytokine production [118]. Alvarez et al. [91] studied the probiotic potential of yeasts isolated from fermented beverages and reported that all yeast strains analyzed were capable of inhibiting flagellin-induced activation of innate inflammatory immune response, with strains from the *Kluyveromyces* and *Pichia* genera being the most immunomodulatory. In another study by Zhu et al. [119], it was shown that yeast-derived β-glucan (from *P. kudriavzevii* DPUL-51–6Y, *Kluyveromyces marxianus* DPUL-F15, and *S. cerevisiae* DPUL-C6 strains) played a significant role in mitigating the inflammatory response and alleviated ulcerative colitis by reshaping the microbial community and metabolite profiles (including indole-3-lactic acid, indole-3-β-acrylic acid, tryptophol, and short-chain fatty acids (acetic, propionic, and butyric acids)) in the host intestinal tract by suppressing NF-κB signaling through the reduction in p65 and IκB-α while simultaneously activating the Nrf2 and AHR pathways.

**Figure 3:**
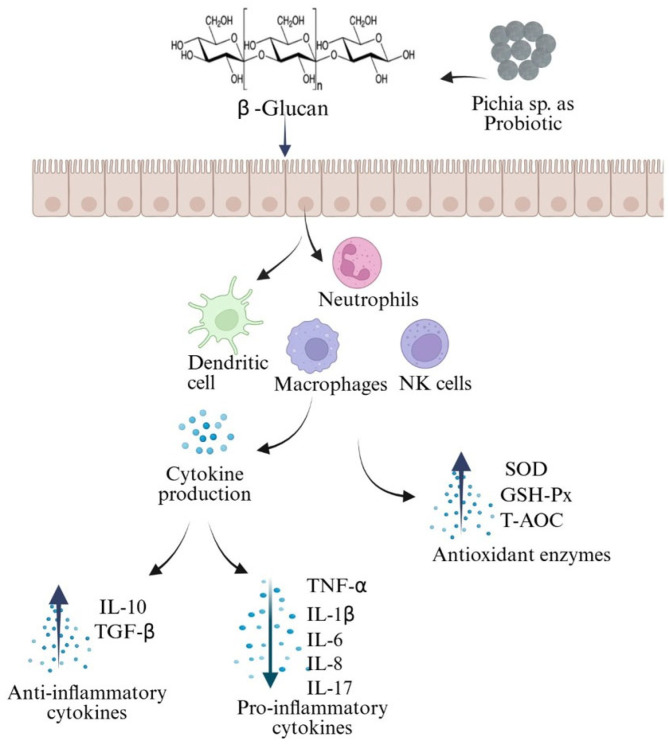
Immunomodulatory properties of *Pichia* increase anti-inflammatory cytokines and antioxidant enzymes and decrease pro-inflammatory cytokines.

Probiotic yeasts interact with immune cells in the gut lining, stimulating the production of cytokines and other immune-mediated proteins like interleukin (IL)-10 and interferon (IFN)-γ, which enhance the body’s overall immune response against invading pathogenic microbes (Figure 3) [120,121]. Numerous studies have documented immune modulation following the administration of *Pichia* species as a probiotic in vivo. For example, in weaned pigs, when *P. anomala* AR2016 was orally administered, it increased mRNA levels of alkaline phosphatase (ALP), toll-like receptors 2 (TLR-2), tumor necrosis factor-α (TNF-α), and interleukin-10 (IL-10) in the jejunal and ileal mucosa and also enhanced the antioxidant defense by upregulating SOD (superoxide dismutase), GSH-Px (glutathione peroxidase) and T-AOC (total antioxidative capacity), thus resulting in improved daily and average growth performance of weaned pigs [103]. When selenium-enriched *P. kudriavzevii* was administered to mice, it downregulated pro-inflammatory cytokines such as TNF-α, IL-1β, IL-6, IL-17 and oxidative stress markers like MPO, MDA, whereas it upregulated the anti-inflammatory cytokine IL-10 and antioxidant enzymes (SOD, CAT, GPX) and suppressed the NF-κB inflammatory pathway [92]. In human colonic epithelial cells, a probiotic yeast, *P. kudriavzevii* (Y1) and its cell-free supernatant (CFS-Y1), downregulated the expression level of pro-inflammatory cytokines such as IL-6, IL-8, and TNF-α, whereas the anti-inflammatory cytokine TGF-β was upregulated. This observation suggests this yeast is more potent in modulating immune responses and could have potential therapeutic benefits for regulating inflammation and enhancing epithelial defense mechanisms in the gut [101]. Moreover, supplementation with *P. kudriavzevii* solid culture considerably reduced the concentration of pro-inflammatory factors (IL-1β, IL-6, IL-8) and increased the concentration of anti-inflammatory factors (IL-10, IL-22) in the small intestines, resulting in improved growth performance of weaned piglets [122]. Together, these results demonstrated the multifaceted role of different *Pichia* species, as it exhibited promising immunomodulatory and probiotic characteristics.

### 4.8. Production of Volatile Organic Compounds

Volatile organic compounds (VOCs) are small flavor-active metabolic products < 300 Da, of organic compounds in living cells, that exhibit a high vapor pressure (vaporization at 0.01 kPa at a temperature of ~20 °C), low solubility in water, but high solubility in lipids [123,124,125]. VOCs such as esters, organic acids, and higher alcohols, determine the characteristic bouquet of fermented products [126]. In addition to enhancing scent, VOCs also include various molecules such as alcohols, thioesters, thioalcohols, cyclohexanes, hydrocarbons, aldehydes, heterocyclic compounds, phenols, ketones, and benzene derivatives that have significant potential against pathogens [127,128,129,130]. Fungal VOCs are derived from primary and secondary metabolism pathways [131]. Many recent studies have indicated that yeast volatiles play an important role in yeast-pathogen interactions. Examples of VOCs produced by probiotic yeast isolates to combat fungal infections include (2-phenylethanol) produced by *P. kudriavzevii* that reduced the growth of *Monascus purpureus* by significantly inhibiting conidium germination and mycelial growth [132]. In another study by Choińska et al. [133], *P. kudriavzevii* and *P. occidentalis* caused more than 50% inhibition of *Penicillium chrysogenum*, *Penicillium expansum*, *A. flavus*, *Fusarium cereals*, *Fusarium poae*, as well as *Botrytis cinerea* by producing ethyl esters of medium chain fatty acids, phenyl ethyl alcohol, and acetate esters. Another *Pichia* species, *Pichia galeiformis*, produced VOCs including ethanol, 3 methyl 1-butanol, phenyl ethyl alcohol, benzaldehyde, benzene acetaldehyde, acetic acid, esters which resulted in 60% inhibition of *Penicillium digitatum* by inhibiting mycelial growth and spore germination [79]. *P. kudriavzevii* MBELGA61 has been used as a biocontrol agent against *Aspergillus* species through the production of soluble and volatile bioactive antifungal compounds like phenylethanol, 2-phenylethyl acetate, and benzyl alcohol [134]. *Pichia membranaefaciens* significantly reduced the rot incidence (percentage of plums that develop brown rot symptoms) produced by *Monilinia fructicola* by inhibiting its spore germination and mycelial growth and by producing volatile compounds [135]. Masoud et al. [136] demonstrated that *P. anomala* and *P. kluyveri* strongly inhibited the growth of *Aspergillus ochraceus* by producing different volatile compounds during coffee processing including ethyl acetate, isobutyl acetate, 2-phenyl ethyl acetate, ethyl propionate and isoamyl alcohol. Taken together, these studies indicate the antifungal potential of VOCs produced by different probiotic species of *Pichia*.

## 5. *Pichia* in Health

*Pichia* is also gaining attention in health and disease research. As the understanding of the human microbiome deepens, the role of *Pichia* in promoting health and preventing disease is becoming a dynamic area of scientific study. For example, *P. kudriavzevii* YS711 demonstrated the ability to degrade uric acid by 31.2% within 24 h, facilitated by a complete uric acid metabolic pathway [36]. This ability may prove beneficial to humans as it has preventative effects against chronic kidney disease and tumorigenesis previously linked to uric acid build-up [36,137,138,139]. In an in vitro study by Ezekiel et al. [140], four *P. kudriavzevii* strains were found to have a 97% gut colonization potential due to their adherence to hydrocarbons, auto-aggregation, and co-aggregation. Moreover, these strains have been able to decrease free radicals, as shown in a decrease of 31 mycotoxins by 1-87% in a 48 h period [140]. Rahbar Saadat et al. [141] investigated the inhibitory role of exopolysaccharides (EPSs) of *P. kudriavzevii* on different colon cancer cell lines and found that the EPSs induced apoptosis in colon cancer cell lines (SW-480, HT-29, HCT-116) by upregulating pro-apoptotic genes (BAX, Caspase-3, Caspase-8) and downregulating the anti-apoptotic gene Bcl-2. Additionally, they suppressed key cancer-promoting signaling pathways, including AKT1, JAK1, and mTOR, with minimal toxicity to normal cells. Although *Pichia* EPSs upregulated antioxidant regulator Nrf-2, it did not induce reactive oxygen species (ROS) production, ferroptosis, or alter glutathione and iron levels, suggesting a selective, non-toxic anticancer mechanism which highlighted the potential of *P. kudriavzevii* EPSs as safe, effective postbiotic agents in gut health and colon cancer prevention.

Saber et al. [142] studied the anticancer activity of *P. kudriavzevii* AS-12 secretion metabolites against human colorectal cancer cell lines (HT-29 and Caco-2) and reported that methanolic extract of *P. kudriavzevii* AS-12 supernatant significantly inhibited proliferation and induced apoptosis through an increase (BAD, CASP-3, CASP-8, CASP-9, and Fas-R) or decrease (Bcl-2) in expression level of pro-/anti-apoptotic genes in the human colon cancer cells (HT-29 and Caco-2). In another study by Ma et al. [103], oral administration of *P. anomala* isolated from traditional solid wine koji and its effects on the growth and health of weaned pigs showed that oral administration of *P. anomala* AR2016 enhanced the growth performance by improving the microflora through increasing beneficial bacteria like *Lactobacillaceae*, *Bacteroidetes*, and *Lachnospiraceae*, while reducing harmful bacteria levels such as *Clostridiaceae* in weaned pigs, enhancing the intestinal barrier function and reducing the incidence of diarrhea. Accumulating evidence shows that *Pichia* spp. exhibits a wide range of beneficial effects in both health and disease contexts. Collectively, these findings highlight the versatile role of *Pichia* spp. in promoting health, modulating microbiota, and contributing to the prevention and potential treatment of various diseases.

## 6. Potential *Pichia* Species as Probiotics

Research advancements and substantial screening efforts have emphasized the probiotic potential of diverse *Pichia* species (Table 3). A literature review showed that numerous strains exhibit key probiotic characteristics including cholesterol assimilation, acid and bile tolerance, hydrophobicity, auto-aggregation, antimicrobial activity, and are safe in in vivo studies. *P. kudriavzevii*, *P. guilliermondii*, *P. manshurica*, *P. fermentans*, *P. norvegensis*, and *P. kluyveri* have all exhibited promising results in a variety of investigations, both in vitro and in food-based systems. These findings show that members of the *Pichia* genus can tolerate GI conditions while also contributing to host health, either directly through bioactive metabolite synthesis or indirectly through gut microbiota modification. *Pichia* species constitute an important and underexplored category of functional food and biotherapeutic applications as interest in non-conventional probiotic yeasts develops. According to FAO/WHO recommendations, a preliminary in vitro assessment is required before evaluating the probiotic qualities of different microorganisms, such as their ability to survive during passage through the GI tract and to ensure they also tolerate bile.

**Table 3:** Summary of probiotic attributes and industrial uses of selected *Pichia* strains.

*Pichia* Strain	Benefits	Reference
*P. anomola* AR2016	Decreases incidence of diarrhea Increases relative abundance of *Bacteroidetes*, *Lachnospiraceae*, and *Succinivibrionaceae* in pig cecum Decreases relative abundance of *Proteobacteria*, *Clostridiaceae*, *Campylobacteraceae*, *Vibrionaceae*, *Bacillus*, and *Pseudon* in pig cecum Increases intestinal digestive enzyme activity and improves digestion and absorption of intestinal nutrients in pigs Increases concentration of tight junction proteins occludin and ZO-1, enhancing intestinal barrier function	[103]
*P. anomala* NCYC 432	Secretes killer toxin that inhibits growth of pathogenic *Candida* species	[143]
*P. guilliermondii* 25A	Moderate antioxidant activity	[144]
*P. kluyveri* LAR001	Antimicrobial activity against *L. monocytogenes*, *P. aeruginosa*, *S. aureus*, *E. coli*, and *Klebsiella* species	[145]
*P. kudriavzevii* GBT37	Proteolytic and lipolytic activity which facilitates digestion Antimicrobial activity against *S. aureus*, *B. cereus*, EPEC K1.1, and *Listeria* species No antibiotic resistance genes No hemolytic activity	[84]
*P. kudriavzevii* HJ2	Produces short-chain fatty acids (SCFAs) High antioxidant capacity Contains no antimicrobial resistance genes or virulence genes Produces extracellular hydrolases that increase nutrient availability	[78]
*P. kudriavzevii* M28	Produces folate and phytases to increase nutritional quality of foods	[85]
*P. kudriavzevii* TS2	Produces phytase to increase nutritional quality of foods No hemolytic activity and non-pathogenic	[11]
*P. kudriavzevii* YGM091	High antioxidant capacity Produces extracellular hydrolases that increase nutrient availability No hemolytic activity	[80]
*P. kudriavzevii* YS711	Degrades uric acid No virulence factors associated with human pathogenesis	[36]
*P. kudriavzevii* Y33	Strong antimicrobial activity against *S. typhi*, *E. coli*, *Shigella*, *P. aeruginosa*, *B. cereus*, *S. aureus*, *A. hydrophila*, and *L. monocytogenes* Decreases blood cholesterol levels through cholesterol assimilation Produces extracellular hydrolases that increase nutrient availability No hemolytic activity	[70]
*P. manshurica* 2A	High catalase activity which is linked to antioxidant capacity	[144]
*P. manshurica* PB54	Antimicrobial activity against *Salmonella enterica* Anti-inflammatory capacity in intestinal epithelial cells	[91]
*P. norvegensis* NYI	Antifungal activity against *A. flavus* Aflatoxin-reducing activity towards aflatoxin B1 (AFB1) and B2 (AFB2)	[108]
*P. occidentalis* GBT30	Proteolytic activity which facilitates digestion Antimicrobial activity against *S. aureus*, *B. cereus*, EPEC K1.1, and *Listeria* species No hemolytic activity	[84]
*P. pastoris* X-33	Inhibits *S. typhimurium* growth Reduces adhesion of pathogenic bacteria to intestinal cells	[109]

## 7. Gaps in the Literature Regarding *Pichia*

We have summarized promising evidence supporting the probiotic potential of *Pichia* and particularly its safety profile. Most of the available evidence comes from preclinical studies, including both in vitro and animal model experiments, which have elucidated mechanisms by which *Pichia* strains may benefit the host. However, several significant gaps must still be addressed before *Pichia* can be reliably established as a probiotic for human consumption.

The most evident limitation in the literature is the lack of clinical safety research involving human subjects. Current findings gathered from preclinical data make it difficult to predict how these benefits will translate to human health outcomes. Additionally, even though certain mechanisms of action have been described, it is important to recognize that not all *Pichia* strains necessarily function in the same way. Thus, more research is needed to distinguish and characterize strain-specific differences in both safety and efficacy studies designed to identify the most suitable strains for health applications.

Another notable gap is the lack of evidence-based recommendations regarding dosage regimens and duration of use. At present, there is insufficient high-quality data to inform practical guidelines on how much *Pichia* should be administered, in what form, and for how long in order to confer expected health benefits. Well-designed and controlled clinical trials will be able to establish the effectiveness and safety of *Pichia* as a probiotic for human consumption and also confirm its safety in diverse human populations. This will provide value to regulatory agencies, industrial developers and consumers.

Beyond clinical research, formulation and regulatory challenges represent further barriers to the widespread adoption of *Pichia* as a probiotic. Technical issues around the development of stable, effective commercial products such as optimizing conditions, maximizing shelf life, and designing delivery systems that ensure high viability and activity of *Pichia* strains, still need to be addressed. Furthermore, while *P. kluyveri* has garnered some regulatory recognition; most other species within the genus have not, due to limited research and safety data. It is critical to deepen our understanding of the attributes and potential of other *Pichia* species so that a broader range of strains can be considered for safe and effective use.

## 8. Conclusions

The genus *Pichia* is increasingly recognized as a promising group of probiotic yeasts, offering physiological and functional beneficial characteristics that set it apart from the more widely used bacterial probiotics. *Pichia* species exhibit remarkable resilience to GI stressors, such as low pH and bile salt conditions, which enhances their survival and adherence within the gut. There are also many strains that demonstrate robust auto-aggregation and adhesion characteristics, which are crucial for effective colonization and for providing a barrier against pathogens. In addition to these traits, several *Pichia* species can assimilate cholesterol, produce a range of bioactive metabolites, and display significant antimicrobial and anti-inflammatory activities. These mechanisms contribute to improved gut epithelial barrier integrity, upregulation of tight junction proteins, support for mucosal healing, and attenuation of inflammation. Most importantly, the majority of tested *Pichia* strains display a favorable safety profile for being non-pathogenic.

Several *Pichia* strains have emerged as leading candidates for further research and probiotic application due to their compelling probiotic attributes. Within the species *P. kudriavzevii*, strain Y33 is distinguished by its high cholesterol assimilation, strong survival under gut-like conditions such as low pH and bile salts, superior auto-aggregation, broad-spectrum antimicrobial activity, and a favorable safety profile [70]. Similarly, *P. kudriavzevii* GBT37 exhibits notable proteolytic, lipolytic, and antimicrobial functions while demonstrating no antibiotic resistance or hemolytic activity [84]. Another *P. kudriavzevii* strain, HJ2, combines the production of short-chain fatty acids with antioxidant activity and confirmed safety, lacking virulence factors and resistance genes [78]. Additional *P. kudriavzevii* isolates, including O21, O26, SH55, and O12, display strong GI resilience, metabolic versatility, heat tolerance, auto-aggregation, and safety [14]. In the species *P. anomala*, strain AR2016 has been shown to markedly improve gut barrier function by upregulating tight junction proteins, increasing intestinal digestive enzyme activity, enriching beneficial gut microbiota, reducing diarrhea, and lowering populations of potentially pathogenic bacteria [103]. Lastly, *P. occidentalis* strain GBT30 possesses proteolytic activity, effective antimicrobial properties, and a favorable safety profile, making it another strong candidate for probiotic development [84].

While numerous *Pichia* strains show potential as food-related probiotics, major challenges remain, including the requirement for complete genetic and functional characterization of the strain under development to meet next-generation probiotic criteria. Validating strain-specific advantages, conducting effective human clinical studies, optimizing dosage regimens, and dealing with formulation and regulatory challenges are among the top research priorities. Since some *Pichia* strains can act as opportunistic pathogens in immunocompromised patients and have been linked to hospital-acquired invasive candidiasis, rigorous strain selection and comprehensive safety assessment are needed when considering *Pichia* for probiotic applications. Successful completion of these research efforts will enable an informed decision on the ability of a given *Pichia* strain to be used as a reliable, validated, and broadly accepted probiotic yeast.

## Data Availability

No new data were created or analyzed in this study. Data sharing is not applicable to this article.

## Abbreviations

GI	Gastrointestinal
GRAS	Generally Regarded as Safe
ISAPP	International Scientific Association for Probiotics and Prebiotics
EFSA	European Food Safety Authority
IBS	Irritable bowel syndrome
TJ	Tight junction
ZO	Zonula occluden
IBD	Inflammatory bowel disease
DSS	Dextran sodium sulfate
LPS	Lipopolysaccharide
IL	interleukin
IFN	interferon
ALP	Alkaline phosphatase
TLR-2	Toll-like receptors 2
TNF-α	Tumor necrosis factor-α
T-AOC	Total antioxidative capacity
GSH-Px	Glutathione peroxidase
SOD	Superoxide dismutase
CFS-Y1	Cell-free supernatant
VOC	Volatile organic compound
